# Bacteriophage-mediated biofilm control: a novel targeted strategy for the management of dental caries

**DOI:** 10.3389/fcimb.2026.1880231

**Published:** 2026-07-28

**Authors:** Huijie Sun, Kaiwen Zhang, Fujiang Chu, Jiajia Dong, Song Chen, Biao Ren, Qiang Guo

**Affiliations:** 1 State Key Laboratory of Oral Diseases & National Center for Stomatology & National Clinical Research Center for Oral Diseases, West China Hospital of Stomatology, Sichuan University, Chengdu, China; 2 Department of Orthodontics Dentistry, West China Hospital of Stomatology, Sichuan University, Chengdu, China; 3 Haoyikang Biotech (Guangzhou) Co., Ltd., Guangzhou, China; 4 Department of Pulmonary and Critical Care Medicine, State Key Laboratory of Respiratory Health and Multimorbidity, West China Hospital, Sichuan University, Chengdu, Sichuan, China

**Keywords:** *Actinomyces*, bacteriophage, biofilm, caries, oral phageome, phage therapy, *Streptococcus mutans*

## Abstract

Dental caries, a globally prevalent chronic infectious disease, is driven by microbial dysbiosis of dental biofilm, involving multiple caries-associated colonizers. Conventional broad-spectrum antimicrobials and fluoride-based formulations often disrupt the commensal oral microbiota, contributing to antimicrobial resistance and imbalance of the oral ecosystem. In contrast, bacteriophage (phage) therapy has emerged as a promising precision strategy for caries control due to its high specificity, minimal off-target effects, and biofilm-disrupting capacity. Our review systematically summarizes recent advances in phage-based interventions for caries prevention and treatment. It describes the diversity and distribution of the oral phageome and its role in regulating oral microbial homeostasis. The review further details phages targeting key cariogenic bacteria, including *S. mutans* (e.g., φAPCM01, SMHBZ8), *Actinomyces* spp. (e.g., Av-1), and related phage enzymes. Core anti-bacterial and anti-biofilm mechanisms of oral phage are elucidated, such as direct bacterial lysis mediated by endolysins and holins, and inhibition and disassembly of biofilms through multiple mechanisms. Strategies for applying phages in caries management are also discussed, encompassing phage cocktails, combination therapies with conventional antimicrobials, and development of advanced delivery systems. Finally, current challenges and future directions of phage therapy are addressed. Overall, this review provides a comprehensive theoretical foundation for developing targeted, oral microbiome-friendly phage-based strategies against dental caries.

## 1 Introduction

Dental caries is a prevalent chronic polymicrobial infectious disease affecting the mineralized structures of teeth (Du et al., 2021). The progression of caries could worsen or trigger systemic diseases, substantially impairing general health, and thus impose significant socioeconomic burden (Kassebaum et al., 2017; Cheng et al., 2022; Spatafora et al., 2024). Dental biofilm composed of oral microbes and surrounded by extracellular matrix is regarded as an important cause of caries. Microbial interactions, horizontal gene transfer of antimicrobial resistance determinants, the complexity of matrix, and the physical protection conferred by exopolysaccharides (EPS) increase the risk of caries (Chen et al., 2020). Consequently, targeting biofilms is crucial for designing and developing effective caries-preventive agents.

Early approaches to managing caries-associated microbiota primarily relied on broad-spectrum antimicrobials and fluoride-based formulations. However, agents such as kanamycin and chlorhexidine can inhibit extensive swaths of the overall microbiota including beneficial organisms. Consequently, the treated site becomes susceptible to outbreaks of drug-resistant pathogens or to being repopulated by detrimental microbial community (Spatafora et al., 2024). Therefore, current research emphasizes developing targeted therapies that selectively eliminate cariogenic microorganisms while preserving the surrounding microbiome to reconstitute a symbiotic microbial community (Marsh, 2018). To achieve this goal, bacteriophage (phage) therapy has become an attractive strategy due to its ability to specifically inhibit caries-related microorganisms. Phages constitute the major component of oral viral community. They can infect oral microorganisms and reshape the oral microbial ecosystem (Banar et al., 2024), which endows them with considerable promise as potent antimicrobials. In addition to individual phages, therapeutic approaches also include phage-derived enzymes, combinations of phages with antibiotics, engineered phages, and multi-phage cocktails for polymicrobial infections (Azam et al., 2021; Martínez et al., 2021). Moreover, phage therapy has the following advantages compared to conventional antimicrobials: high specificity for particular strains and low impact on the commensal flora; replicating locally at the site of infection and disappearing with the target pathogen; effective in destroying biofilms; minimal toxicity; and able to be genetically engineered (Betts et al., 2013; Torres-Barceló and Hochberg, 2016; Baker et al., 2017; Steier et al., 2019).

In the field of common oral diseases, phages targeting periodontal pathogens (Matrishin et al., 2023; Xu et al., 2025) and endodontic pathogens (Khalifa et al., 2016) have been predicted or isolated. Phage-based particle vaccines have shown promise in preventing oral infections linked to human papillomaviruses, which are related to head-and-neck cancers (Guo et al., 2024). Similarly, various phages against cariogenic bacteria like *Streptococcus mutans* (*S. mutans*) (Dalmasso et al., 2015; Ben-Zaken et al., 2021), and *Actinomyces* were reported and found to lyse these microorganisms or inhibit their biofilms, which offers potential candidates for therapeutic applications of caries (Delisle et al., 1978; Shlezinger et al., 2017; Lv et al., 2023; Spatafora et al., 2024).

This review aims to systematically summarize the research progress on phages in caries prevention and treatment. We elaborate on the oral phageome, summarize phages targeting major cariogenic bacteria, elucidate their biological mechanisms of anti-bacterial and anti-biofilm action, and discuss phage therapy and its potential applications in caries management. By highlighting its potential as an ecologically targeted strategy, this work seeks to provide a theoretical foundation and research perspective for developing targeted, oral microbiome-friendly anti-caries strategies.

## 2 Oral phageome: diversity and characteristics

The oral cavity, one of the human body’s most microbially diverse habitats, harbors abundant bacteriophages, collectively termed the oral phageome. These phages play key roles in regulating the oral microbiome’s composition and function, and show substantial diversity in taxonomy, spatial distribution, and functional potential, closely linked to oral health and diseases like dental caries (De La Cruz Peña et al., 2018; Szafrański et al., 2021; Chen et al., 2024a).

### 2.1 Composition and distribution of oral phageome

The oral phageome is dominated by tailed phages of the order Caudovirales (96% of isolates), primarily *Siphoviridae*, *Myoviridae*, and *Podoviridae*. *Siphoviridae* and *Myoviridae* prevail in healthy individuals, while non-tailed phages (e.g., *Microviridae*) are detected in minor proportions (De La Cruz Peña et al., 2018; Szafrański et al., 2021; Rybicka and Kaźmierczak, 2025).

Oral phages are distributed across all microhabitats: saliva, a primary reservoir with up to 10^5^ virus-like particles/μL, facilitates phage dispersal (Edlund et al., 2015; Szafrański et al., 2021); dental plaque harbors phages targeting caries-associated bacteria (Chen et al., 2024a; Chen et al., 2024b); and mucosal surfaces/mouthwash samples host unique communities (Chen et al., 2024a; Jie et al., 2025). Their host range centers on six dominant oral bacterial phyla, with Streptococcus-infecting phages most linked to caries (Szafrański et al., 2021; Chen et al., 2024b; Chen et al., 2024a).

### 2.2 Oral phage databases and “dark matter”

Comprehensive databases advance phage research: the Oral Phage Database (OPD) contains 189,859 non-redundant sequences (including 3,416 huge phages), and the Oral Virus Database (OVD) adds 48,425 viral sequences (Li et al., 2022; Jie et al., 2025). However, “viral dark matter” —uncharacterized phages and unknown-function proteins —persists: 78.71% of OPD-encoded proteins lack annotations, and most abundant oral phages remain uncultured. Integrated methodological frameworks, such as metagenomics combined with single-virus genomics (SVGs), have proven effective in unraveling the cryptic diversity of the oral phageome—exemplified by vSAG 92-C13, a prominent *Streptococcus*-infecting phage ranked among the most abundant taxa in human oral viromes (De La Cruz Peña et al., 2018; Jie et al., 2025). Furthermore, emerging artificial intelligence (AI)-driven and structural biology approaches are now being harnessed to decode this “viral dark matter” and address the long-standing challenge of functional annotation of uncharacterized phage proteins. Deep learning-based protein structure prediction tools such as AlphaFold, ESMFold, ColabFold, and FoldSeek have enabled structure-based function inference for proteins with limited sequence homology, capitalizing on the principle that conserved three-dimensional folds may retain functional similarities even when sequence identity is low (Klein-Sousa et al., 2025; Sinno et al., 2025). Fine-tuning of protein language models on viral protein sequences has been shown to significantly enhance representation quality and improve performance on downstream functional annotation tasks, further advancing tools for understanding the vast and understudied viral proteome (Sawhney et al., 2025).

### 2.3 Population specificity and disease association

The oral phageome is population-specific, shaped by geography, urbanization, and lifestyle (Abeles et al., 2014; Jie et al., 2025; Rybicka and Kaźmierczak, 2025). Caries correlates with phageome alterations: Severe early childhood caries (S-ECC) is marked by reduced alpha diversity, increased beta diversity, decreased *Streptococcus* phages, and elevated *Podoviridae* compared to the healthy group (Chen et al., 2024b). The association between phageome alterations and caries is attributed to the dysregulation of phage-bacteria interactions. In healthy oral ecosystem, beneficial phages regulate microbial homeostasis by preying on cariogenic bacteria and preventing their overgrowth. In caries-prone individuals, however, this balance is disrupted: reduced abundance of lytic phages targeting *S. mutans* weakens the inhibitory pressure on cariogenic bacteria, leading to biofilm dysbiosis and acid production (Edlund et al., 2015; Marsh and Zaura, 2017; Chen et al., 2024b). Conversely, lysogenic phages may contribute to caries development by transferring virulence factors (e.g., antibiotic resistance genes) to cariogenic bacteria, enhancing their pathogenicity (Wang et al., 2016; Chen et al., 2024a). These findings underscore the potential of the oral phageome as a therapeutic target and diagnostic tool for dental caries.

## 3 Natural bacteriophages targeting major cariogenic bacteria

Caries-associated microbiota form dense biofilms with EPS as the matrix, enhancing their acid-producing capacity and antimicrobial tolerance, which has become a key barrier that traditional anti-caries strategies struggle to overcome (Sharahi et al., 2019; Osman et al., 2025). In recent years, bacteriophage resources targeting major cariogenic bacteria have been continuously explored, with focus on *S. mutans* and *Actinomyces* spp.

### 3.1 Bacteriophages targeting *S. mutans*

*S. mutans* is widely recognized as a primary cariogenic pathogen, due to its strong acidogenicity, aciduricity, and capacity to produce EPS (Palmer et al., 2019; Alshahrani and Gregory, 2020; Zhang et al., 2020). Over the past decades, multiple *S. mutans*-specific phages have been isolated and characterized for their potential in caries prevention. This section reviews five representative bacteriophages with a focus on their isolation sources, host ranges, key genomic or functional features, and biofilm-inhibitory effects (**Table 1**).

**Table 1:** Core features and anti-bacterial activity of natural phages targeting *Streptococcus mutans*.

Phage	Isolation source	Host range (susceptible/total tested)	Genome size	Life cycle	effective MOI	Biofilm inhibition	Key reference(s)
M102	Human dental plaque	c: 7/7 e:0/2 f: 0/2	31,147 bp	Lytic	unknown	unknown	(Delisle and Rostkowski, 1993; Shibata et al., 2009)
M102AD	Human oral cavity	c: 1/25	30,664 bp	Lytic	0.1	unknown	(Delisle et al., 2012)
φAPCM01	Human saliva	c:0/13 e:1/4	31,075 bp	Lytic	lower than 2.5x10^-3^	Yes (At doses of 10^5^–10^9^ pfu/well: OD_492nm_ values approaching that of the medium control; viable cells decrease ≥5 log CFU/ml at 24 h)	(Dalmasso et al., 2015; Fu et al., 2017)
SMHBZ8	Samples of saliva, teeth, dental plaques, dental sewage, and regular sewage	c: 13/13 e:0/2 f:0/2	32,460 bp	Lytic	0.1	Yes (At dose of 10^8^ pfu/well: OD_595nm_ reading reduce three-fold at 24 h, 4.8-fold at 48 h, 6.8-fold at 72 h)	(Ben-Zaken et al., 2021; Wolfoviz-Zilberman et al., 2021)
φKSM96	Clinical S. mutans isolates	c: 102/105 e: 11/14 k: 0/4 f: 0/2	39,820 bp	Temperate	unknown	Yes (At dose of 2 × 10^10^ and 2 × 10^9^ pfu/well suppressed biofilm formation by 80% and 30% at 24 h, respectively)	(Sugai et al., 2023)

MOI, Multiplicity of Infection; pfu, plaque-forming unit; CFU, Colony Forming Unit.

#### 3.1.1 M102 and M102AD

First isolated by Tiraby’s group in 1988, M102 is one of the most well-characterized lytic phages targeting *S. mutans* (Delisle and Rostkowski, 1993). Its genome comprises 31,147 base pairs with a GC content of 39.21%, belonging to the family *Siphoviridae*. Phage M102 may encode two endolysins with distinct substrate specificities (e.g., muramidase, amidase, or endopeptidase)—a relatively uncommon feature, as most phages typically combine multiple specificities within a single polypeptide (Van Der Ploeg, 2007). M102 exhibits strict dependence on rhamnose–glucose polysaccharide (RGP) side chains for host recognition, conferring high specificity for *S. mutans* serotype c strains (Shibata et al., 2009).

M102AD was isolated from the oral cavity and shared 90.8% genomic homology with M102 at the nucleotide level. Unlike M102, it carries a unique small open reading frame (ORF), *orf40*, whose production lacks homology to any known proteins. M102AD exhibits slow adsorption (1.5×10^-10^ ml/min), and its host range remains narrow, infecting only 1 out of 25 tested *S. mutans* isolates (Delisle et al., 2012).

#### 3.1.2 φAPCM01

φAPCM01, a novel phage isolated from human saliva after screening 85 samples, belongs to the *Siphoviridae* family with B1 morphology. It also exhibits a narrow host range, specifically targeting *S. mutans* serotype e strain. Its genome is 31,075 bp with a 39% G-C content, containing 37 ORFs (23 with putative functions). Biochemically, φAPCM01 shows exceptional anti-bacterial activity: short latent period (60 min) and burst size (~44 phage particles/cell) enable efficient propagation within biofilms, leading to sustained reduction in metabolic activity and viable cell counts of bacteria. φAPCM01 remains effective at extremely low multiplicity of infection (MOI), making it suitable for low-dose applications in oral formulations (Dalmasso et al., 2015; Fu et al., 2017).

#### 3.1.3 SMHBZ8

Isolated from 254 diverse samples (saliva, teeth, dental plaques, and sewage), SMHBZ8 is a promising candidate for caries-preventive phage therapy. Morphologically classified as a member of the *Siphoviridae* family (Caudovirales order, B1 morphology), it displays strict host specificity, exclusively targeting *S. mutans* serotype c. Genomically, it has a 32,460 bp genome (38.8% G+C content), sharing 76-77% sequence identity with related phages (M102, M102AD, φAPCM01) and carrying 32 unique genes—including two lysin-encoding genes that may enhance lytic activity (Ben-Zaken et al., 2021). SMHBZ8 exhibits potent biofilm-inhibitory efficacy in an animal study: it reduces preformed *S. mutans* biofilms and prevents carious lesions in caries models with efficacy comparable to chlorhexidine (Wolfoviz-Zilberman et al., 2021).

#### 3.1.4 φKSM96

Phage φKSM96, a temperate bacteriophage isolated from clinical *S. mutans* strains, exhibits remarkable potential for caries control. It belongs to the *Siphoviridae* family and possesses a circular double-stranded DNA genome of 39,820 bp. It displays broad host specificity for *S. mutans* across different serotypes, exerting selective anti-*S. mutans* activity without adsorbing to other oral bacteria (e.g., *S. sanguinis*, *S. salivarius*, *S. mitis*). *In vitro* studies confirm its significant inhibition of planktonic *S. mutans* growth and biofilm formation. Notably, unlike virulent phages, φKSM96 does not induce strong lysis and shows only modest particle proliferation in one-step growth curve assays, suggesting bactericidal activity via endolysin or holin post-adsorption (Sugai et al., 2023). As the first isolated temperate phage targeting *S. mutans*, its unique lysogenic property provides a valuable foundation for genetic manipulation to enhance anti-bacterial efficacy, addressing the limitations of previously reported lytic phages.

### 3.2 Bacteriophages targeting *Actinomyces* species

*Actinomyces* spp. stand as key etiological agents of root caries, with *A. naeslundii* and *A. gerencseriae* consistently identified in carious lesions (Bowden et al., 1999; Brailsford et al., 1999; Dame-Teixeira et al., 2016). However, in stark contrast to the relatively well-characterized phage resources for *S. mutans*, reports on *Actinomyces*-associated bacteriophages remain extremely scarce and outdated. Phages targeting these species were first isolated from human dental plaque samples. Four such isolates—AV-1, AV-2, AV-3, and 1281—exhibit binding affinity for *A. viscosus* of coaggregation group A as well as *A. naeslundii* of coaggregation group E. Phages AV-1, AV-2, and 1281 display lysis curves characteristic of productive infection. Phage AV-1 generates distinct clear plaques (2–3 mm in diameter) when plated on *A. viscosus* MG-1 lawns, and its host specificity is limited strictly to serotype 2 strains of *A. viscosus* (Delisle et al., 1978). A subsequent study in 1997 isolated additional phages and established transfection methods, but these efforts focused on genetic tool development rather than anti-caries applications and still lacked genomic sequencing or *in vivo* evaluation (Yeung and Kozelsky, 1997). Critically, no modern isolation, genomic characterization, or preclinical evaluation of *Actinomyces*-specific phages has been reported. Unlike the situation for *S. mutans*—where multiple lytic phages have been genomically sequenced, tested in biofilm and animal models, and considered for therapeutic development—the evidence base for *Actinomyces* phages remains rudimentary and purely descriptive. This profound disparity represents a critical research gap that urgently requires attention. Given the established etiological role of *Actinomyces* spp. in root caries, future efforts should prioritize isolating novel lytic phages targeting contemporary clinical strains, followed by genomic characterization, biofilm disruption assays, and animal model validation. Without such investment, phage therapy for caries will remain incomplete in addressing the full polymicrobial nature of the disease.

## 4 Biological mechanisms of bacteriophages against cariogenic bacteria and dental biofilms

**Figure 1:**
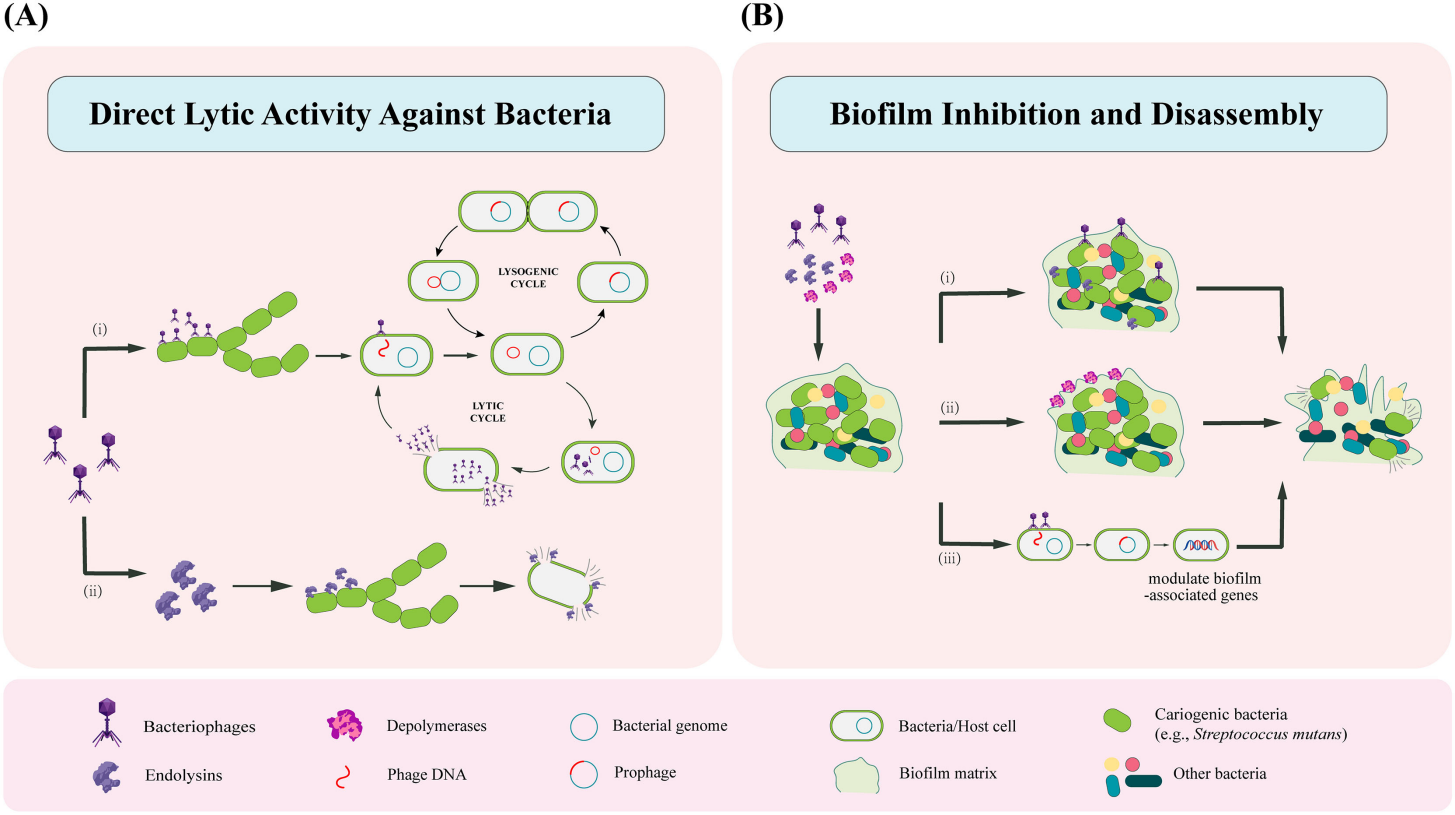
Dual mechanisms of bacteriophages against cariogenic bacteria and dental biofilms. **(A)** Direct lytic activity against planktonic cells of cariogenic bacteria. (i) Bacteriophages bind to host receptors, inject genomic DNA, and initiate either lytic replication or lysogenic integration. Phage-encoded holins and endolysins act from within to hydrolyze the peptidoglycan in cell wall, ultimately lysing the bacteria. (ii) Phage endolysins specifically hydrolyze peptidoglycan bonds, disrupting cell wall integrity and triggering rapid osmotic lysis. **(B)** Inhibition and disassembly of biofilms. (i) Phages infiltrate the biofilm matrix to infect and eliminate cells of EPS-producing bacteria and other cariogenic bacteria through similar mechanisms summarized in part **(A)**. (ii) Phage-encoded depolymerases and engineered lysins degrade biofilm matrix. (iii) Phages integrate into the bacterial genome and impair EPS synthesis and biofilm assembly via modulating the transcription of biofilm-associated genes.

Bacteriophages exhibit two core mechanisms to weaken the pathogenicity of cariogenic pathogens: direct lysis of host bacterial cells and disruption of biofilm integrity via degrading or impairing the synthesis of biofilm matrix (**Figure 1**). These mechanisms leverage the high specificity of phages, minimizing disruption to the commensal oral microbiota while targeting pathogenic strains (Osman et al., 2025).

### 4.1 Direct lytic activity against cariogenic bacteria

The anti-bacterial activity of phages against cariogenic bacteria is rooted in their specific infection and lytic processes. Phages first recognize and adsorb to host-specific receptors on the surface of target bacteria via tail structures, which confers their species/strain specificity. Stable adsorption triggers local degradation of the bacterial peptidoglycan layer by tail-associated glycosidases, enabling the injection of phage genomic DNA into the host cytoplasm (Inamdar et al., 2006; Leiman and Shneider, 2012) (**Figure 1A**). Post genome injection, phages follow two distinct life cycles. Strictly lytic phages then commandeer the host’s biosynthetic machinery to produce progeny virions, culminating in cell lysis mediated by phage-encoded holins and endolysins (**Figure 1A**). By contrast, temperate phages integrate into the host genome as prophages, thereby modulating bacterial physiology and biofilm-related behavior, and may switch to the lytic cycle in response to environmental signals (Erez et al., 2017; Harada et al., 2018) (**Figure 1A**). Phage-induced host lysis in Gram-positive bacteria depends on a holin-endolysin system. Holins form timed pores in the cytoplasmic membrane to allow endolysins to reach and degrade the peptidoglycan cell wall (**Figure 1A**). For Gram-negative bacteria, an additional protein, spanin, is required to mediate inner and outer membrane fusion during cell lysis (Young et al., 2000; Cahill and Young, 2019; Abdelrahman et al., 2021).

Numerous *in vitro* studies have demonstrated that endolysins exert potent anti-bacterial activity against a range of Gram-positive pathogens, with *S. mutans* being a primary target in the context of caries control. The temperate bacteriophage φO1205 harbors a lysis module containing *lyt*51, which encodes the lytic enzyme Lyt51. This enzyme specifically targets the cell walls of *S. mutans* and *Streptococcus thermophilus* by recognizing the lysine-alanine_2-3_ interpeptide bridge7 in the peptidoglycan layer, and mediates their *in vitro* lysis (Sheehan et al., 1999). *Streptococcus gordonii* OMZ1039 (isolated from supragingival plaque) harbors the inducible *Siphoviridae* prophage PH15. Its purified endolysin, expressed in *Escherichia coli*, inhibits cariogenic *S. mutans* and other pathogenic *streptococci*, alongside *S. gordonii* strains (Van Der Ploeg, 2008). Zhao’s group also showed that LysP53, a bacteriophage lysin originally targeting *Acinetobacter baumannii*, exhibits selective activity against *S. mutans*—even in the acidic carious microenvironment (pH 4.0-6.5). It reduces viable bacterial counts in *S. mutans* biofilms and demonstrates comparable efficacy to 0.12% chlorhexidine in preventing enamel demineralization (Zhao et al., 2023).

### 4.2 Inhibition and disassembly of biofilms

Under natural conditions, phages are shaped by natural selection to survive and compete with other phages, and their interactions with biofilm-associated bacteria are analogous to those with planktonic bacteria in many respects (Abedon, 2015). Phages replicate and release increasing numbers of infectious progeny that spread through the biofilm matrix, eliminate cells of EPS-producing bacteria, as well as other cariogenic bacteria, progressively remove the biofilm and reduce its regeneration potential (**Figure 1B**). They can also infect persister cells and remain dormant within inactive cells until reactivation to initiate productive infection and cell destruction (Harper et al., 2014). Naturally occurring phages isolated from oral specimens demonstrate promising biofilm-degrading capabilities. Phage φAPCM01 effectively inhibits *S. mutans* biofilm formation and growth in a dose-dependent manner. High doses (10^5^-10^9^ pfu/well) achieve complete metabolic inhibition and substantial reduction in viable cells, with sustained efficacy over 48 h (Dalmasso et al., 2015). Phage SMHBZ8 achieves nearly complete inhibition of *S. mutans* biofilm formation over a 72-h period, with the 24-h OD reading of treated samples being three times lower than that of the control, and the bacteria in the treated biofilm appeared dispersed and lacked intercellular connections (Ben-Zaken et al., 2021). Similarly, 80% and 30% suppression of *S. mutans* (10^7^ cells/mL) biofilm formation were observed following the addition of φKSM96 at doses of 2 × 10¹^0^ PFU/well and 2 × 10^9^ PFU/well, respectively (Sugai et al., 2023).

Many phage genomes carry genes encoding depolymerases which decompose biofilm matrix components—typically targeting the host cell wall upon phage release and possessing the capacity to degrade biofilm EPS when liberated from lysing cells (Yan et al., 2014) (**Figure 1B**). EPS components vary across bacterial species, yet given that EPS exhibits lower variability compared to the host bacteria themselves, enzymes tend to display broader functional activity (Harper et al., 2014). Furthermore, engineered biotechnological approaches enable the expansion or modification of lysins’ host range while boosting their anti-biofilm efficacy. Bioengineered chimeric lysin, ClyR—engineered by fusing the CHAP catalytic domain (PlyCAC) from group C *streptococcal* bacteriophage (Nelson et al., 2001) and the cell wall-binding domain (PlySb) from *Streptococcus suis* phage lysin PlySs2 (Gilmer et al., 2013)—shows rapid, broad-spectrum lytic activity against *S. mutans*, with only weak activity against harmless oral commensals (Yang et al., 2015, Yang et al., 2016; Xu et al., 2018). It exhibits potent activity against recalcitrant biofilms: 100 μg/mL ClyR reduced biofilm biomass by over 80%, while 100 μg/mL penicillin exerted no appreciable effect on that of *S. mutans* (Yang et al., 2016). The anti-bacterial activity of ClyR against *S. mutans* is mediated by specific interaction between glucosyltransferases (Gtfs)-derived water-insoluble EPS and ClyR (Ma et al., 2025). This structural specificity in recognition may be analogous to the mechanism by which phage tail spike proteins bind and degrade bacterial surface polysaccharides (e.g., capsular polysaccharides, EPS, lipopolysaccharides) (Lee et al., 2017).

Apart from inducing the host genome to produce EPS-degrading depolymerizing enzymes, recombinant phage could also modulate the transcription of biofilm-associated genes to impair matrix synthesis and structural assembly (**Figure 1B**). Bacteriophage T4 RNA ligase 1 (T4 Rnl1), which can be stably expressed in various bacteria, impairs biofilm formation of *S. mutans* without altering its growth phenotype. Due to T4 Rnl1-mediated alterations in the production and distribution of EPS and downregulated mRNA transcription of biofilm-associated genes (e.g., *gtfB*), the T4 Rnl1 mutant exhibits reduced biofilm mass, a defective EPS matrix, and a sparse, poorly established biofilm architecture (Chen et al., 2021). Interestingly, it has been observed in a variety of bacterial species that phage genes can stably integrate into the host genome as prophages, and conversely promote biofilm formation (Carrolo et al., 2010; Gödeke et al., 2011). A linear plasmid-like prophage xhp1, identified via genomic analysis of *A. odontolyticus* strain XH001, enhances host biofilm formation through spontaneous induction and subsequent release of extracellular DNA, thereby promoting host persistence in the oral cavity (Shen et al., 2018).

## 5 Strategies for applying phages in caries management

Phage-based therapeutic strategies for caries prevention and treatment can be advanced through multiple interrelated approaches.

### 5.1 Cocktail formulations

Dental biofilm involves multiple caries-associated microorganisms with high complexity and interindividual variability. Consequently, mono-phage therapy is often limited by narrow host ranges and the rapid emergence of microbial resistance. Rationally designed phage cocktail therapy that comprises multiple phages targeting different cariogenic microorganisms can broaden antimicrobial coverage and mitigate resistance development. For instance, based on 47 collections of 20 *Enterococcus faecalis* phages, Wandro et al. demonstrated that combining two or more different phages markedly lowered the occurrence of phage-resistant mutants (Marsh, 2018). Likewise, phages specific to cariogenic pathogens such as *S. mutans* and *Actinomyces* spp. can be utilized in combination to achieve enhanced anti-caries efficacy (Steier et al., 2019; Szafrański et al., 2021; Strathdee et al., 2023; Pal et al., 2024).

### 5.2 Combination with antimicrobial agents

Synergistic interactions between phages and conventional antimicrobials offer a promising avenue to potentiate caries control. Phages can disrupt biofilm architecture, thereby improving penetration of co-administered agents such as fluoride, chlorhexidine, or antimicrobial peptides. Conversely, subinhibitory concentrations of certain antimicrobials may suppress bacterial defense systems, thereby enhancing phage infectivity. Moreover, studies in other infection models have demonstrated that the combination of antibiotics and phages can obtain excellent therapeutic effect and resensitize resistant pathogens to antibiotics (Torres-Barceló and Hochberg, 2016; Shlezinger et al., 2019; Canfield et al., 2021; Kim et al., 2024), suggesting a solution that could be adapted to manage dysbiotic cariogenic biofilms.

### 5.3 Advanced delivery systems and formulation design

The dynamic and complicated nature of the oral environment, including salivary flow, pH fluctuations, and robust biofilm matrix, poses significant challenges to phage retention and activity. To overcome these barriers, engineered delivery platforms are essential. Encapsulation of phages in biocompatible hydrogels, nanoparticles, or slow-release films can protect phages from enzymatic degradation and prolong their residence time at local target sites. Topical formulations such as phage-infused mouth rinses or varnishes have also been proposed to facilitate localized delivery. Recently, quite a few encapsulated carriers have been utilized in phage delivery systems targeting the intestinal microbiota (Yang et al., 2023). These systems show advantages in maintaining phage stability, safeguarding infectivity, and enabling controlled release of phages within the colon. While the oral environment differs ecologically from the colonic, phage delivery approaches could similarly be integrated with dental biomaterials to manage or avert caries. Furthermore, future innovations may incorporate stimuli-responsive materials that trigger phage release under cariogenic conditions (e.g., low pH), enabling spatiotemporally controlled intervention precisely when and where acidogenic activity peaks (Guo et al., 2024).

## 6 Challenges and prospects for phage therapy in caries management

### 6.1 Challenges

Many *in vitro* studies have demonstrated the efficacy of phage-based approaches against cariogenic biofilms, and these findings are further corroborated by *in vivo* animal studies using whole phages (e.g., SMHBZ8) or phage-derived lysins (e.g., ClyR), which have shown significant reduction in carious lesion development and enamel demineralization in rodent models, with no detectable cytotoxicity or adverse effects observed (Yang et al., 2016; Xu et al., 2018; Wolfoviz-Zilberman et al., 2021). Despite this promising preclinical evidence, direct data from human clinical trials for phage therapy specifically targeting dental caries remain absent, and several significant challenges must be addressed before clinical translation can be achieved.

The oral cavity itself presents formidable physicochemical barriers to phage therapy. Saliva is continuously secreted at an average rate of 0.3 mL/min, increasing to a maximum of 7 mL/min during mastication or stimulation (Humphrey and Williamson, 2001). This constant flow creates a strong hydrodynamic force that rapidly washes away free phage particles from the tooth surface and dental plaque. pH fluctuation represents another critical challenge to phage stability. Bacteriophages, being proteinaceous entities, exhibit variable pH tolerance; while many maintain infectivity within pH 5.0-10.0, exposure to pH < 4.0 can cause irreversible protein denaturation and complete loss of infectivity (Ben-Zaken et al., 2021; Sugai et al., 2023). While the resting oral temperature is maintained at 36.5-37.5 °C, consumption of hot or cold foods and beverages can cause rapid temperature swings ranging from 0 °C to 70 °C (Barclay et al., 2005). Temperatures > 70 °C cause thermal denaturation of capsid proteins and reduce phage viability (Mrevlishvili et al., 1999).

Beyond mechanical clearance, saliva is biochemically active and contains numerous antimicrobial factors, including secretory IgA (sIgA), lysozyme, lactoferrin, histatins, and mucins—that may directly neutralize or enzymatically degrade phage virions (Rahman et al., 2026). Phage delivery has also been shown to increase the level of sIgA, which may reduce therapeutic efficacy upon repeated administration (Górski et al., 2012; Huang et al., 2021; Li et al., 2024). This concern cannot be dismissed, yet the topical application of anti-caries phages in oral cavity offers a distinct immunological advantage over systemic delivery. Locally administered phages are largely confined to the oral mucosa and dental biofilm, with minimal systemic leakage, thereby substantially reducing the induction of circulating anti-phage IgG/IgM and the risk of systemic inflammatory cascades (Majewska et al., 2019; Gembara and Dąbrowska, 2024). Moreover, the high local phage concentrations may competitively overcome the sIgA barrier, ensuring sufficient phage penetration into the cariogenic biofilms.

The phage resistance mechanisms inherent to *S. mutans* represent another challenge. First, adsorption receptor mutation is the common source of resistance (Shibata et al., 2009; Labrie et al., 2010). Then, restriction-modification systems in *S. mutans* may methylate self-DNA while selectively cleaving improperly modified foreign bacteriophage DNA (Maruyama et al., 2009; Claisse et al., 2026). The genome of *S. mutans* contains two clustered regularly interspaced short palindromic repeats (CRISPR) loci that actively acquire bacteriophage-derived DNA fragments as spacers, thereby protecting the bacteria against phage invasion and contributing to resistance against bacteriophage infection (Van Der Ploeg, 2009; Claisse et al., 2026). The dynamic interplay between these diverse defense systems and bacteriophage evasion strategies drives persistent antagonistic coevolution between bacteriophages and *S. mutans*. Bacteriophage pressure selects for and enriches resistant host mutants, while bacteriophages may sustain infectivity by mutating receptor-binding proteins and modifying bases to evade defense systems of *S. mutans* (Buckling and Rainey, 2002; Koskella and Brockhurst, 2014). The co-evolutionary dynamics implies that single-phage formulations may rapidly lose efficacy in the complex environment of oral cavity. Therefore, to design durable phage-based prevention and treatment strategies for caries, it is essential to systematically consider the above defense mechanisms. For instance, employ phage cocktails targeting distinct receptors, engineer phages to carry anti-CRISPR proteins, or combine adjuvants capable of interfering with restriction-modification systems, to delay the emergence of resistance and enhance the long-term effectiveness of phages in caries control.

Finally, the lack of standardized protocols for phage production, purification, and quality control, combined with the absence of clear regulatory frameworks for phage-based therapeutics in dentistry, remains a major barrier to clinical application (Shlezinger et al., 2017; Steier et al., 2019).

### 6.2 Prospects

Despite the challenges outlined above, phage therapy for caries management holds considerable promise. Encouragingly, while direct human clinical data for caries-specific phage therapy remain absent, the broader field of phage therapy has accumulated substantial clinical experience in other infectious diseases, providing a strong foundation for future translation. A recent meta-analysis of 130 studies (1,115 patients) reported that phage therapy achieved 71% clinical improvement and 51% bacterial eradication in drug-resistant infections, with customized phages showing higher efficacy (79% and 87%, respectively); adverse events were predominantly mild to moderate (Liu et al., 2025). Another systematic review of phage therapy for Gram-positive infections over the past 15 years further confirmed a favorable safety and efficacy profile, highlighting the specificity and selectivity of phage-based interventions (Nie et al., 2025). A range of clinical case series and trials have demonstrated successful applications against multidrug-resistant pathogens in diverse infection settings, including *Pseudomonas aeruginosa*, *Staphylococcus aureus*, and *Mycobacterium abscessus* (Ooi et al., 2019; Chan et al., 2025; Ferreiro-Posse et al., 2026). Collectively, these clinical data demonstrate that phage therapy is both feasible and safe in human patients, establishing a favorable translational precedent for eventual application to dental caries.

Expanding phage resources through OPD and other phage databases will facilitate the discovery of novel lytic phages targeting cariogenic microorganisms and complement existing phage-based interventions against primary caries pathogens (Szafrański et al., 2021; Jie et al., 2025). For example, a variety of virulent and temperate phages infecting *Lactobacilli* spp.—such as Lcb, vB_LcaM_Lbab1, ΦPYB5, LFP02, and LFP03—have already been isolated from dairy products, fermented vegetables, and industrial fermentation tanks (Zhang et al., 2015; Gradaschi et al., 2021; Lv et al., 2023; Zhang et al., 2024), and exhibit potent lytic activity and, in some cases, broad-spectrum anti-bacterial effects (Zhang et al., 2006; Wang et al., 2008; Zhang et al., 2011), thus providing a novel, targeted strategy to selectively deplete these cariogenic *Lactobacillus* populations within dental biofilms, although their actual effects in oral environment need to be further verified.

Deeper mechanistic insights into complex interactions among bacteria, phages, and oral cells within the complicated oral ecosystem are essential for rational therapy design (Guo et al., 2024). Emerging platforms driven by AI promise to accelerate phage identification, host prediction, and engineered optimization (Doud et al., 2025). Deep learning models like PHPGAT can predict phage hosts based on multimodal heterogeneous knowledge graph with graph attention network (Liu et al., 2024; Malajczuk et al., 2026). Moreover, prophage prediction tools such as VirFinder and VirSorter can identify viral sequences from oral metagenomes, contributing to find novel phages for caries-related microorganisms (Roux et al., 2015; Ren et al., 2017). For AI-guided engineering of chimeric endolysins, protein structure prediction tools like AlphaFold3 can optimize the fusion of catalytic domains and cell wall-binding domains from different origins, enabling the rational design of chimeric endolysins with high activity and narrow host spectrum (Jumper et al., 2021). These AI-driven strategies promote shifting phage therapy from empirical screening to intelligent design.

Through synthetic biology, bacteriophages can be purposefully engineered, for example, by introducing biofilm-degrading enzyme genes or expanding host range, to enhance the elimination of caries-related microorganisms within dental plaque. CRISPR-enhanced phages can deliver CRISPR-Cas systems to induce chromosomal double-strand break or precisely target specific sequences in cariogenic bacterial genomes, thereby effectively killing the pathogen or attenuating its cariogenic potential (Wizrah, 2026). Given the high interpersonal variation in oral microbiomes, personalized phage therapy to individual microbial profiles represents a promising precision medicine approach (Banar et al., 2024). Furthermore, it is a promising strategy to suppress caries-related microorganisms while preserving commensals to modulate the microbial composition of dental plaque biofilms using phage therapy, thereby reshaping oral ecosystem homeostasis and achieving ecological caries prevention. These converging frontiers are expected to collectively advance phage therapy from concept to clinical practice in caries management.

## 7 Conclusion

Phage therapy represents a promising precision approach for managing dental caries, enabling highly specific targeting of key cariogenic bacteria while preserving the commensal oral microbiota—thereby helping to restore and maintain oral microbial homeostasis and addressing critical limitations associated with conventional broad-spectrum antimicrobials and fluoride-based interventions. Phages exert anti-caries effects through direct lysis of cariogenic bacteria and multi-faceted disruption of cariogenic biofilms. Development of phage-based therapeutic strategies, including phage cocktails, combination therapies with conventional agents, and advanced delivery systems, further enhance the applicability of phage therapy. Despite challenges for phage therapy in caries management, such as narrow host ranges, safety and stability concerns, regulatory gaps, as well as the lack of support from human clinical trials, ongoing advances in phage resource exploration, mechanistic research, and AI-driven optimization hold great potential for developing personalized, oral microbiome-friendly anti-caries interventions. Future efforts focusing on large-scale clinical trials and standardized protocols will be pivotal to translating phage therapy into routine caries prevention and treatment.
